# Clinical characteristics, echocardiographic findings, and 6-month outcomes in Ugandan women with peripartum cardiomyopathy

**DOI:** 10.1093/eschf/xvaf005

**Published:** 2026-01-08

**Authors:** Juliet Nabbaale, Karen Sliwa, Annettee Nakimuli, Graham Chakafana, Wanzhu Zhang, Peter Lwabi, John Omagino, Sulaiman Lubega, Elias Sebatta, James Kayima, Emmy Okello

**Affiliations:** Uganda Heart Institute, Upper Mulago Hill, Mulago Hospital Complex, Kampala, Uganda; Division of Cardiology, Department of Medicine, Faculty of Health Sciences, Cape Heart Institute, University of Cape Town, Cape Town, South Africa; Division of Cardiology, Department of Medicine, Faculty of Health Sciences, Cape Heart Institute, University of Cape Town, Cape Town, South Africa; Department of Obstetrics and Gynaecology, School of Medicine-Makerere University, College of Health Sciences, Kampala, Uganda; Department of Chemistry and Biochemistry, Hampton University, Hampton, VA, USA; Uganda Heart Institute, Upper Mulago Hill, Mulago Hospital Complex, Kampala, Uganda; Uganda Heart Institute, Upper Mulago Hill, Mulago Hospital Complex, Kampala, Uganda; Uganda Heart Institute, Upper Mulago Hill, Mulago Hospital Complex, Kampala, Uganda; Uganda Heart Institute, Upper Mulago Hill, Mulago Hospital Complex, Kampala, Uganda; Uganda Heart Institute, Upper Mulago Hill, Mulago Hospital Complex, Kampala, Uganda; Uganda Heart Institute, Upper Mulago Hill, Mulago Hospital Complex, Kampala, Uganda; Department of Medicine, School of Medicine-Makerere University, College of Health Sciences, Kampala, Uganda; Uganda Heart Institute, Upper Mulago Hill, Mulago Hospital Complex, Kampala, Uganda; Division of Cardiology, Department of Medicine, Faculty of Health Sciences, Cape Heart Institute, University of Cape Town, Cape Town, South Africa; Department of Medicine, School of Medicine-Makerere University, College of Health Sciences, Kampala, Uganda

**Keywords:** Peripartum cardiomyopathy, Heart failure, Global longitudinal strain, Africa

## Abstract

**Introduction:**

Peripartum cardiomyopathy (PPCM) affects previously healthy women commonly of African ancestry resulting into elevated morbidity and mortality rates. The clinical characteristics of PPCM are diverse but there is yet limited data on outcomes for women with PPCM in Uganda. We sought to elucidate the clinical presentation, echocardiographic findings, and 6-month outcomes among women with PPCM in Uganda.

**Methods:**

A prospective cohort study of 80 PPCM women matched for age were monitored over a 6-month period while on goal-directed medical therapy (GDMT) was conducted. All participants underwent a physical examination, 12-lead electrocardiography, echocardiography and biomarkers including NT-pro BNP and Prolactin at baseline and at 6-month follow-up visit. Additionally, 80 matched controls were recruited at baseline as comparison for the biomarkers.

**Results:**

The mean age of cases and controls was 33.6 ± 6.6 and 30.2 ± 5.9 years respectively. Clinical data for cases were as follows: mean left ventricular ejection fraction (LVEF) was 35.7 ± 11.0%, mean LV global longitudinal strain (GLS) was −11.9 ± 4.7%, mean right ventricular GLS was −14.7 ± 10.9%. A total of 22 (27.5%) participants had a LVEF <35% while 6 (7.5%) participants had severe RV systolic dysfunction. 20 (25%) participants were in NYHA IV. 54 (68%) participants received bromocriptine therapy in addition to other GDMT. Clinical data for controls were as follows: mean LVEF was 67.2 ± 4.5%, mean LV GLS was −17.1 ± 4.9%, all controls had normal RV systolic function parameters. After 6-months of follow-up, 6 (7.5%) of the cases had died. Atrial fibrillation occurred in 2 (2.5%) participants and intracardiac thrombus was documented among 8 (10%) participants. 52 (65%) participants were in NYHA I. LV recovery (LVEF ≥ 50%) was observed in 37 (46.3%) cases.

**Conclusion:**

This study shows a high mortality rate of 7.5% aligning with global studies, the observed high thrombus burden and stroke occurred in 10% and 2.5%, respectively which may indicate severity of LV systolic dysfunction at presentation. Two-thirds of patients received Bromocriptine in addition to GDMT which may explain the high rate of LV recovery in this cohort.

## Introduction

Peripartum cardiomyopathy (PPCM) is a potentially life-threatening *de novo* cause of heart failure that is defined as a cardiomyopathy secondary to left ventricular (LV) systolic dysfunction towards the end of pregnancy or in the months following delivery, where no other cause of heart failure is found.^1^ PPCM is a global disease that affects previously healthy women and is associated with variation in the clinical presentation, adverse outcomes such as thromboembolism, arrhythmias, persistent LV systolic dysfunction and death. Peripartum cardiomyopathy therefore presents a significant problem due to its high morbidity and mortality rates globally.^2^

The global burden of PPCM has been shown to vary with the highest incidence of PPCM reported in Nigeria (1:102 live births)^3^ and Haiti (1:299 live births).^4^ In South Africa, there was more than one incident case of PPCM for every 1000 live births^5^ compared with the Americans where it is reported as 1 case per 3000–4000 live births.^4,6^ The accurate burden of PPCM in Africa remains less well-established, primarily due to lack of population-based studies. One of the most recent studies conducted by our group showed that the prevalence of PPCM in Uganda was 17.4%, with incomplete LV recovery (LVEF < 50%) observed in 22 participants (53.7%).^7^ However, there is still limited knowledge regarding other adverse outcomes, particularly mortality and thromboembolic events among patients with PPCM in Uganda.

At the global stage, understanding of the aetiology of PPCM also remains limited although various risk factors have been associated with PPCM over the years.^1^ The proposed risk factors include advanced maternal age, multiparity, pre-eclampsia, multiple pregnancy, obesity, poor health expenditure, selenium deficiency and African ancestry.^1,8–10^

The objective of this study therefore, was to describe the clinical presentation, echocardiographic findings and the outcomes at 6-months of follow-up amongst women with PPCM in Uganda.

## Methods

This was a single centre, prospective cohort study conducted from June 2022 to August 2023. Participants were screened at the outpatient clinic of the Uganda Heart Institute (UHI), the antenatal and postnatal clinics of Kawempe National Referral Hospital. UHI is a national referral heart hospital in Kampala, Uganda offering specialized adult and paediatric medical and surgical cardiology services. The institute has a Siemens Artis Q biplane cardiac catheterization laboratory and several echocardiography machines including a GE E95 and Siemens Acuson Origin that are able to perform global longitudinal strain (GLS) tests. Participants were enrolled into the study after obtaining written informed consent. Ethical approval for the study was obtained from the respective Ethical Research Committees of Makerere University (School of Medicine Research Ethics Committee) with IRB approval number Mak-SOMREC-2022-287 and University of Cape Town (Higher Research Ethics Committee) with IRB approval number HREC 093/2022. The study conformed to the ethical guidelines of the Declaration of Helsinki on the principles for the medical research involving human subjects.^11^

### Peripartum state

This is defined as the last month of pregnancy and first 6-months post-delivery.

### Study population

Inclusion criteria for cases comprised:

- Peripartum state
- Manifestation of signs and/or symptoms of heart failure;
- Left ventricular (LV) ejection fraction <45%
- Exclusion of alternative causes of heart failure

Exclusion criteria for cases:

- A peripartum woman with heart failure confirmed with an identifiable cause.

Inclusion criteria for controls:

- Peripartum state
- Matched for age and weeks into puerperium
- No signs and/or symptoms of heart failure

Exclusion criteria for controls:

- Inability to consent

### Sample size estimation

A study by Blauwet *et al*. showed that using RV FAC at presentation predicted LV recovery and clinical events in patients with PPCM. They further estimated that the standard deviation (SD) of the baseline RV FAC was 11.2, therefore based on these estimates we determined that at 5% level of significance and 80% power would require 80 participants to detect a 5-unit difference in RV FAC that ranges between 5 and 10 units. This sample size was obtained using formula of hypothesis testing for two population means.

### Study procedure

#### Echocardiography protocol and equipment

A Vivid E95® (GE Healthcare) echocardiography machine was used to acquire the cardiac images by two cardiologists with a 1.5–4.6 MHz adult transthoracic transducer (M5Sc). All images were stored and analysed by a single observer. LVEF was assessed by 2D method from apical four- and two-chamber views, calculated by ‘automatic EF’ function. Further manual adjustment was done whenever necessary. TAPSE and MAPSE were measured by M-Mode at the apical four-chamber view. Strain analysis with STE was achieved by applying automatic function imaging (‘AFI’ function). Left ventricular global and regional longitudinal systolic strain were measured through 3 different views (apical three-, four-, and two-chamber views). Manual adjustment was attempted, in the case of unsatisfied endocardial tracking. All the regional longitudinal strains were demonstrated on a Bull’s eye diagram, while GLS was calculated as the mean of the regional longitudinal strains.

**Figure 1:**
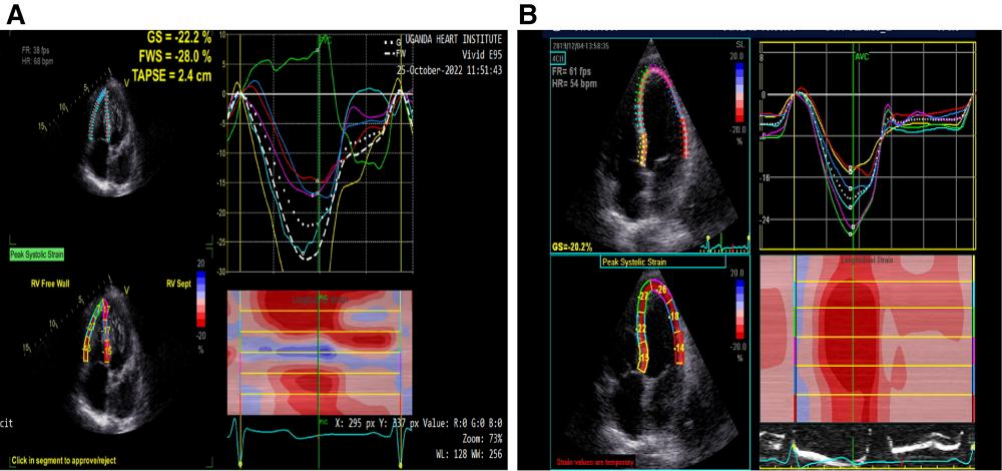
(A) Shows an example of a participant with abnormal left ventricular systolic function parameters (left ventricular global longitudinal strain −2.2%) at baseline. (B) Shows the same participant with full left ventricular systolic function recovery parameters (left ventricular global longitudinal strain −20.2%) at 6-months follow-up

Right ventricular systolic function assessment was performed using the following modalities; right ventricular end-diastolic area (RV EDA) and RV end-systolic area (RV ESA) were measured with manual tracing of digital images from the apical four-chamber view. RV fractional area change (RV FAC) was calculated as follows: RV FAC (%) = (RV EDA -RV ESA)/RV EDA X 100.^12^ RV longitudinal free wall and global wall strain were assessed with speckle-tracking echocardiographic analysis and measurements were performed online with dedicated software (2D Cardiac Performance Analysis). Peak longitudinal systolic strain was obtained for the basal, middle and apical segments of the RV free wall and interventricular septum from software generated curves and averaged for mean RV free wall and RV global strain values. *Figure 1A and B*.

#### Study variables

The baseline visit was the first visit to the specialist making the diagnosis of PPCM. A pretested questionnaire was used to collect data both at baseline and 6-month follow-up which included social demographic characteristics, medical history, obstetric history, clinical examination findings and data from electrocardiography, echocardiography and information on goal-directed medical therapy, anticoagulation and bromocriptine. Blood samples were taken at enrolment for prolactin, N-terminal pro-B-type natriuretic peptide (NT-pro BNP ), complete blood count (CBC), liver function tests (LFTs), renal function tests (RFTs), random blood sugars (RBS) at the 6-month follow-up visit as per protocol.

Over a 6-month period, enrolled PPCM cases were monitored while on appropriate heart failure medication; women in the pre-partum period received diuretics and beta-blockers while women who were in the post-partum period received diuretics, ACE inhibitors or angiotensin receptor blocker, beta-blockers, and mineralocorticoid receptor antagonist. Additionally, bromocriptine therapy (2.5 mg twice daily for 14 days and 2.5 mg once daily for 6 weeks) with anticoagulant therapy at prophylactic dose was administered.

#### Follow-up data

Additional outcomes reported at 6-months following diagnosis of PPCM included death, stroke, thromboembolic events and LV function at 6-months. LV function at 6-months was categorized as: (i) LV recovery defined as presence of LVEF >50%; (ii) persisting moderate LV dysfunction (defined as LVEF 36%–49%); persisting severe LV dysfunction (defined as LVEF <35%).

#### Biomarkers

Blood samples were taken at enrolment for prolactin, NT-pro BNP, CBC, LFTs, RFTs, RBS, and at the 6-month follow-up visit as per protocol.

### Statistical analysis

Normally distributed continuous data was summarized as mean plus or minus SD while skewed data was summarized as median with corresponding interquartile range. Categorical data was summarized as frequencies and for categorical variables with more than two categories, proportions were presented in tables and graphs. We determined the relationship between independent variables and PPCM with a background study design of a nested case-control at bivariate analysis using a Pearson Chi-square test or Fisher’s exact test for categorical variables, a two-sample *t*-test or Wilcoxon rank sum for continuous variables. A *P*-value of <.05 was considered statistically significant. Assumptions of logistic regression were tested and we dropped variables with collinearity which was assessed with a variance inflation factor (VIF) >10 and 1/VIF <0.10. All variables with *P* < .2 were further assessed at multivariate analysis for risk factors for PPCM using logistic regression to assess odds ratios with their 95% confidence intervals and *P*-values. A chunk test was done to assess for interaction. Factors with *P* < .05 will be considered significant. Confounding was also assessed between the crude and adjusted models and a 10% change or more was considered significant. The statistical software analysis version used was SPSS v17.0.

## Results

### Description of study participants

The baseline characteristics of the 80 PPCM cases enrolled are presented in *Table 1*. The mean age of the cases was 33.6 ± 6.6 years, mean parity was 3.6 ± 2.1 pregnancies, mean heart rate was 90.4 ± 23.6 bpm, mean systolic blood pressure was 114.3 ± 11.7 mmHg.

**Table 1:** Sociodemographic and obstetric characteristics of the cases and controls

Variables	Cases(*N* = 80)	Controls (*N* = 80)	*P*-value
**Demographic characteristics**			
**Age in years, mean** **±** **SD**	33.6 ± 6.6	30.2 ± 5.9	.353
**Body mass index**	27 ± 7.0	25.4 ± 6.6	.581
**Body surface area**	1.8 ± 0.4	1.8 ± 0.3	.012
**Weeks post**-**postartum**	9.5 ± 6.5	9.2 ± 5.9	.023
**Occupation status**			.507
**Employed**	50(48.1)	54(51.9)	
**Education status**			.582
**No education**	7(58.3)	5(41.7)	
**Primary education**	22(57.9)	16(42.1)	
**Secondary education**	21(44.7)	26(55.3)	
**Tertiary education**	30(47.6)	33(52.4)	
**Living location**			.011
**Urban**	67(55.8)	53(44.2)	
**Rural**	13(32.5)	27(67.5)	
**Obstetric history**			
**Parity**	3.6 ± 2.1	2.7 ± 1.6	.021
**Gravidity**			.068
**1**	19(54.3)	16(45.7)	
**2–4**	50(54.9)	41(45.6)	
≥**5**	11(32.3)	23(67.6)	
**Number of twin pregnancies**	0.1 ± 0.3	0.8 ± 0.3	.045
**Weeks at onset of heart failure**	9.5 ± 6.5		.076

At baseline, 14 (17.5%) cases were in New York Heart Association (NYHA) Class I, 31 (38.8%) were in NYHA Class II, 15(18.5%) were in NYHA Class III, 20 (25%) were in NYHA Class IV. The onset of heart failure occurred within 9.5 ± 6.5 weeks post-delivery accompanied by dyspnoea as the predominant symptom in 80% of cases as represented in *Table 1*. The mean left ventricular ejection fraction (LVEF) at baseline was 35.7 ± 11.0%. 27.5% of the cases had LVEF <35%, mean LV GLS of −11.9 ± 4.7%, mean RV FAC was 32.9 ± 13.5%, mean RV GLS and −14.7 ± 10.9%.

Six months follow-up data included; (i) death occurred in 6 (7.5%) women and the cause of death was cardiovascular in all cases; of these, (66.7%) were due to heart failure and 33.3% were due to sudden death, (ii) thromboembolic events and stroke occurred among 8 (10%) and 2 (2.5%) of the participants respectively, despite receiving anticoagulation therapy. All strokes were ischaemic. Atrial fibrillation was documented among 2 (2.5%) participants. Figty-two (65%) participants were in NYHA Class I, 1 (1.3%) was in NYHA Class IV.

### Pharmacological treatment

**Figure 2:**
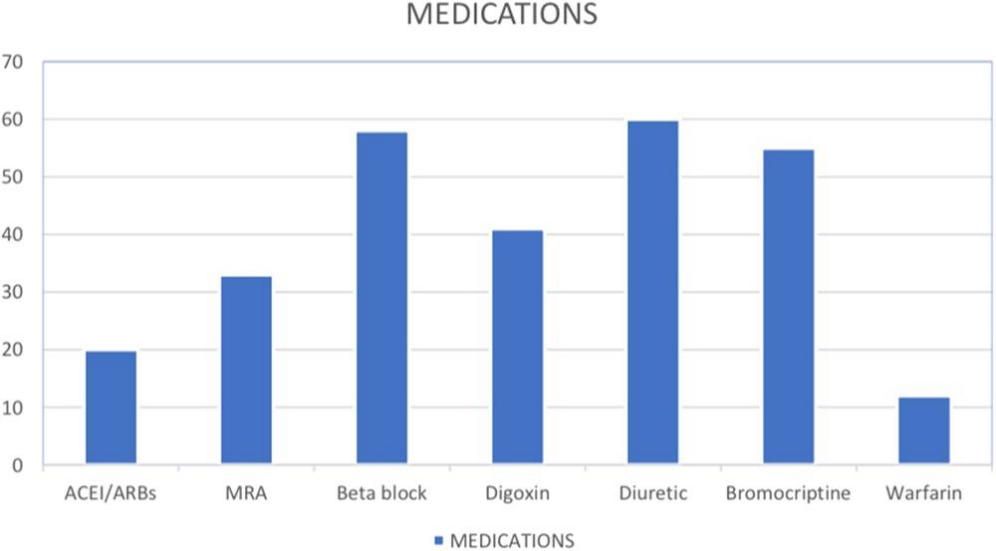
Shows a bar graph of prescription patterns using standard medical therapy for heart failure treatment with beta-blockers, Angiotensin receptor blockers, Lasix, MRAs, digoxin, warfarin. The use of optimal goal-directed medical therapy for heart failure was relatively high; A total of 20 (25%) patients were prescribed either angiotensin-converting enzyme inhibitors or angiotensin receptor blockers, 33 (41.3%) were prescribed spironolactone, 58 (72.5%) were prescribed beta blockers. Digoxin was used in 41 (51.3%) and a diuretic in 60 (75.0%). A total of 68.7% (55) were prescribed Bromocriptine, 15% (12) participants were prescribed Warfarin

The utilization of optimal goal-directed medical therapy for heart failure is demonstrated in *Figure 2* that showed that almost two-thirds were on beta-blockers and almost quarter of the cases (*n* = 22, 27.5%) were prescribed angiotensin receptor blockers, a third (*n* = 33, 41.3%) were prescribed spironolactone. 58 (72.5%) were prescribed beta blockers. Digoxin was used in 41 (51.3%) and a diuretic in 60 (75.0%). A total of 68% (54) were prescribed bromocriptine, 12.5% (10) participants were prescribed Warfarin due to its availability without cost implications compared with the none-vitamin K antagonist oral anticoagulants.

### Maternal outcomes at 6-months follow-up

**Figure 3:**
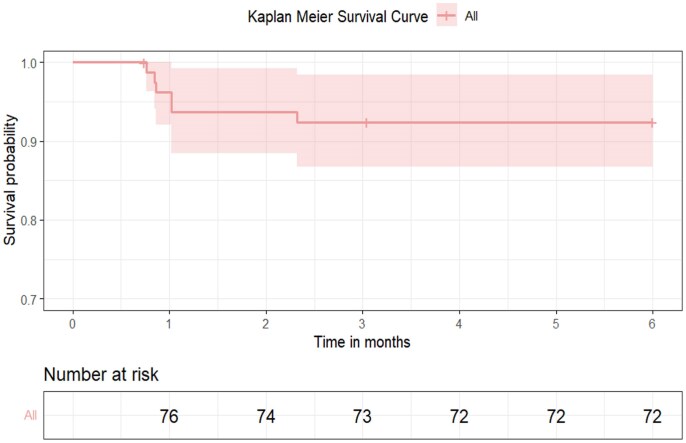
Kaplan–Meier survival curve showing probabilities of the 80 cases over the 6-months’ time period of follow-up with a 3 months survival probability of 92.4%

The 6-month Kaplan–Meier survival curve probabilities of the 80 cases is shown in *Figure 3*. 2 (2.5%) participants were lost to follow-up and 6 (7.5%) participants died. A total of 10 (12.5%) of the participants received anticoagulation for atrial fibrillation (*n* = 2, 2.5%) or intracardiac thrombus (*n* = 8, 10%). 2 (2.5%) participants developed stroke. 1 (1.25%) participants had an ICD inserted for intractable ventricular tachycardia, 52 (65%) participants were in NYHA Class 1.

LV recovery (LV EF > 50%) occurred in 37 (46.3%) participants, there was an absolute increase in LVEF from 35.7 ± 11.0% to 45.9 ± 15.7% (*P* < .001) which was accompanied by a reduction in LV end-systolic diameter from 6.1 ± 0.7 cm to 5.5 ± 1.0 cm (*P* < .001).

At the end of the 6-months, 14 (31.8%) participants had moderate LV systolic dysfunction and 23 (51.1%) had severe LV systolic dysfunction as represented in *Table 2* with additional parallel improvement in NYHA class from 25% of cases in NYHA Class IV at baseline to 1.3% at 6-months in *Table 3*.

**Table 2:** Echocardiography findings in cases at baseline and 6-months follow-up

Characteristic	Baseline *n* (%)	6 months n (%)	*P*-value
**Left ventricular function**			
**LVEF**			**<**.**001**
**Normal**	0	37 (46.3)	
**Mildly abnormal**	28 (35)	0	
**Moderately abnormal**	30 (37.5)	14 (17.5)	
**Severely abnormal**	22 (27.5)	23 (28.8)	
**LV GLS**			.**017**
**Normal**	13 (16.3)	15 (18.8)	
**Mildly reduced**	17 (21.3)	30 (37.5)	
**Moderate**	32 (40)	15 (18.8)	
**Severe**	18 (22.5)	14 (17.5)	
**Right ventricular function**			
**TAPSE**			.**006**
**Normal**	21 (26.3)	35 (43.9)	
**Mild**	0	0	
**Moderate**	14(17.5)	9 (11.3)	
**Severe**	45 (56.3)	27 (33.8)	
**RV Lat S’**			.**155**
**Normal**	20 (25)	26 (32.5)	
**Moderate**	39 (48.8)	37 (46.3)	
**Severe**	21 (26.3)	11(13.8)	
**RV GLS, %**			.**012**
**Normal**	34 (42.5)	44 (55)	
**Mildly reduced**	18 (22.5)	20 (25)	
**Moderate**	17 (21.3)	4 (5)	
**Severe**	11 (13.8)	6 (7.5)	

LVEF, left ventricular ejection fraction; LV GLS, left ventricular global longitudinal strain; TAPSE, tricuspid annular plane systolic excursion; RV Lat S’, right ventricular lateral S wave; RV GLS, right ventricular global longitudinal strain.

Grading for the severity of each parameter is as follows: (i) LVEF: Normal ranges 54% to 74%, mildly abnormal 41% to 53%, moderately abnormal ranging from 30% to 40% and severely abnormal below 30%. (ii) Global longitudinal strain (GLS) normal parameters > −18% to −26%, mildly reduced −15% to −12.5%, moderately reduced ranging −8.1% to −12.5% and severe < −8.0%. Categorical distribution of right ventricular systolic function parameters included (i) TAPSE: Normal 17–25 mm, mildly reduced 16–20 mm, moderately reduced 15–12 mm, and severely reduced was below 10 mm. (ii) Lateral S’: Normal ≥10 cm, mildly impaired 9–12 cm/s, moderately impaired 10–15 cm/s, and severely reduced Lat S’ ranging from 6 to 9 cm/s. (iii) Right ventricular GLS; normal −35% to −17%, mildly reduced −16% to −12%, moderately reduced −11% to −8%, and severely reduced <−7%.

**Table 3:** Clinical, laboratory and ECG characteristics of the cases at baseline and 6 months

Characteristic	Baseline	6 months	*P*-value
**Systolic BP (mmHg)**	115.9 ± 13.3	115.0 ± 11.3	.644
**Diastolic BP (mmHg)**	70.8 ± 8.6	71.0 ± 6.7	.880
**Heart rate (bpm)**	86.5 ± 23.1	78.7 ± 19.3	.026
**BMI (kg/m^2^)**	26.5 ± 6.0	26.9 ± 5.6	.637
**NHYA**			.05
**I**	14(17.5%)	52(65%)	
**II**	31(38.3%)	17(21.3%)	
**III**	15(18.8%)	3(3.8%)	
**IV**	20(25%)	1(1.3%)	
**Laboratory investigations**			
**WBC (10^9^/l)**	6.2 ± 2.3	5.3 ± 1.7	.008
**Hb (g/dl)**	13.5 ± 1.6	13.3 ± 1.5	.394
**MCV (Fl)**	85.6 ± 7.2	86.7 ± 7.4	.411
**Sodium (mmol/l)**	134.6 ± 2.4	136.5 ± 3.8	<.001
**Potassium (mmol/l)**	4.2 ± 0.7	4.1 ± 0.4	.384
**Chloride (mmol/l)**	105.8 ± 4.4	107.1 ± 6.4	.129
**Creatinine (mg/dl)**	0.90 ± 0.3	0.81 ± 0.3	.064
**Urea (mg/dl)**	25.4 ± 13.4	21.5 ± 7.6	.032
**GFR (ml/min)**	103.9 ± 43.8	117.5 ± 33.3	.062
**NT-pro BNP (pg/ml)**	1269.7 (154.5–3787.2)	141.6 (24.5–1126.8)	.078
**Prolactin (ng/ml)**	63.6 ± 35.5	38.1 ± 50.1	.003
**CRP (mg/l)**	0.45 (0.15–1.10)	0.37 (0.08–2.05)	.244
**ECG**			
**PR interval (ms)**	155.6 ± 42.8	165.9 ± 34.5	.104
**QRS (ms)**	88(81–97)	88(81–98)	.745
**Atrial fibrillation**	2(2.5%)	0	
**Ventricular tachycardia**	1(1.25%)	0	
**QTc (ms)**	424.3 ± 46.8	395.4 ± 23.3	**<**.**001**
**LBBB**	3.4(4.25%)	0	

BP, blood pressure; SD, standard deviation; BMI, body mass index; NYHA, New York Heart Association; WBC, white blood cell count; Hb, haemoglobin; MCV, mean cell volume; ECG, electrocardiography; LBBB, left bundle branch block; mmHg, millimetres of mercury; bpm, beats per minute; kg/m^2^, kilogram per square metre; g/dl, grams per decilitre; mmol/l, milimoles per litre. All parameters of the characteristics are represented as mean ± SD.

**Table 4:** Multivariate analysis for factors associated with PPCM

Characteristic	Crude OR (95%CI)	Adjusted OR (95%CI)	*P*-value
Demographic characteristics			
Age categories			
<25	1.00	1.00	
25–34	1.20 [0.47–3.05]	1.04 [0.24–4.46]	.954
35+	3.95 [1.47–10.61]	**5.92 [1.35–25.95]**	.**018**
Weeks post-partum			
Puerperium	1.00	1.00	
Post puerperium	0.45 [0.22–0.94]	**0.22 [0.07–0.69]**	.**010**
Clinical factors			
Potassium	2.41 [1.17–4.99]	**8.58 [1.97–37.32]**	.**004**
Sodium	0.64 [0.53–0.76]	0.78 [0.61–1.01]	.057
Chloride	0.77 [0.69–0.86]	**0.81 [0.70–0.94]**	.**006**
QRS interval	1.03 [1.01–1.06]	**1.05 [1.01–1.10]**	.**029**
Heart rate	1.06 [1.04–1.09]	**1.07 [1.04–1.10]**	**<**.**001**

## Discussion

In this prospective study designed to evaluate the clinical, echocardiographic, laboratory and 6-months outcomes among PPCM patients in Uganda, we found that although there was a high baseline burden of morbidity and mortality among the cases, we also documented a high percentage of LV recovery and improvement in NYHA class. Specifically, 65% of cases improved to NYHA class I functional staging at the end of the 6 months follow-up period. These findings are almost consistent with findings from the EORP global registry where improvement to NYHA Class 1 occurred among 67% of cases. This improvement in clinical status was backed up by improvement in echocardiographic and laboratory biomarker levels at the end of the 6-months follow-up. This dramatic improvement in clinical status is attributable to the natural history of the disease, close access to health care, optimization of GDMT and administration of bromocriptine that further yielded improvement in LV systolic dysfunction and reduction in the mortality. Similarly, data from a systematic review and meta-analysis of 593 patients with PPCM demonstrated that bromocriptine was associated with significantly higher survival rates and greater improvement in LVEF.^13^

Whereas improvement of LV function and size have been shown to occur among 40% of a selected cohort of patients with idiopathic cardiomyopathy,^14^ in this study we found that LV recovery occurred among 46.3% of the cases which is similar to the data from the EORP PPCM registry where LV recovery defined as an improvement in LVEF > 50% occurred in 46% of cases at 6 months.^2^ To the best of our knowledge, this is one of the highest rates of LV recovery reported in a group of patients with PPCM receiving optimized goal-directed medical therapy and administration of bromocriptine, although direct comparison with other cohorts is limited by variation in the definition of recovery, and lack of available treatment data.

Data from a single cohort of PPCM women with severely reduced baseline LVEF ≤ 25% showed poorer outcomes compared with patients with baseline LVEF >25% irrespective of whether bromocriptine was introduced to the standard of care heart failure therapy.^15^

However, we documented lower rates of prescription of standard heart failure therapy with findings of low prescription patterns of ACE Inhibitors, ARBs and Spironolactone compared with the global EORP data despite a significant percentage who presented in advanced heart failure stage.

However, the high use of beta-blockers was similar to that seen in the IPAC study.^16^ Under the EORP registry, the use of diuretics and digoxin were more commonly prescribed in patients of Africa origin which coincided with findings from our pharmacological data. Findings from our study further support our assertions that improvements in cardiac function occurs on goal-directed medical therapy and our data further coincides with findings from the investigations of pregnancy-associated cardiomyopathy (IPAC) study in North America, in which 72% of women recovered by 1 year,^16^ however participants in the IPAC study were followed up for 1 year.

Lower LV recovery rates were documented from the Nigerian PEACE registry in which only 24% of women recovered by 1 year.^17^ Our study is limited by variation in the parameters in the definition of LV recovery, a short follow-up period of 6-months may not fully capture long-term outcomes, and the single centre design may limit the generalizability of the findings.

There was a relatively high burden of mortality at 7.5% which is similar to the documented EORP global registry mortality rate at 6% and this was attributed to the late onset of symptoms in this cohort of patients that contributed to delayed diagnosis compounded by PPCM patients presenting in advanced disease stage. In this cohort, onset of symptoms occurred within 9.5 ± 6.5 weeks post-delivery whereas the internationally known median time from time of onset to diagnosis was 10 days ranging from 6 days in Europe to 23 days in Africa.^2^

Systemic thromboembolic events occurred among 10% of participants and these were characterized by either venous thromboembolism or cerebral vascular accidents that occurred among 2.5%.

The high thrombus burden is likely due to the procoagulant state of pregnancy and the advanced systolic dysfunction in this cohort. Globally, EORP reported a frequency of 9% in Europe, 3% in Asia-Pacific which implies further clinical trials are needed to support the indications for anticoagulation beyond intracardiac thrombus, atrial fibrillation or severely reduced systolic function of the left ventricle. It is therefore important from that therapeutic anticoagulation in PPCM women should be emphasized as per guidelines in addition to goal-directed medical therapy as per guidelines.

Larger cohort studies of consecutive peripartum patients are ideal in Uganda to determine the incidence of PPCM in Uganda.

### Limitations of the study

This study had a short follow-up period of 6 months that may not have enabled us to fully capture the long-term outcomes therefore requiring another study with a longer follow-up period to provide more informative data about the clinical predictors and foetal outcomes. Additionally, this study was a single centre study design whose findings might not be representative of the general population in Uganda hence recommending for larger multicentred cohort studies to determine the incidence of PPCM In Uganda.

## Conclusion

In this cohort of Ugandan patients with PPCM, there was a high mortality rate of 7.5% aligning with global studies, the observed high thrombus burden and stroke occurred in 10% and 2.5%, respectively which may indicate severity of LV systolic dysfunction at presentation. Two-thirds of patients received Bromocriptine in addition to GDMT which may explain the high rate of LV recovery in this cohort.

## Declarations

### Data Availability

No data were generated or analysed for this manuscript.

### Pre-registered Clinical Trial Number

None supplied.
